# Endoscopic Submucosal Dissection Outcomes for Gastroesophageal Tumors in Low Volume Units: A Multicenter Survey

**DOI:** 10.1155/2016/5670564

**Published:** 2016-11-07

**Authors:** Ahmad Najib Azmi, Christopher J. L. Khor, Khek-Yu Ho, Rapat Pittayanon, Rungsun Rerknimitr, Thawee Ratanachu-ek, Doreen S. C. Koay, Jianyi Calvin Koh, Shiaw-Hooi Ho, Khean-Lee Goh, Sanjiv Mahadeva

**Affiliations:** ^1^Division of Gastroenterology, Department of Medicine, University Malaya Medical Center, Kuala Lumpur, Malaysia; ^2^Faculty of Medicine and Health Sciences, Universiti Sains Islam Malaysia, Malaysia; ^3^Department of Gastroenterology & Hepatology, Singapore General Hospital, Singapore; ^4^Department of Gastroenterology, National University Hospital, Singapore; ^5^Division of Gastroenterology, Department of Medicine, Chulalongkorn University Hospital, Bangkok, Thailand; ^6^Department of Surgery, Rajavithi Hospital, Bangkok, Thailand

## Abstract

*Background and Aims.* Endoscopic submucosal dissection (ESD) outcomes have traditionally been reported from high volume centers in East Asia. Data from low volume centers in other parts of Asia remain sparse.* Methods.* A retrospective survey with a structured questionnaire of 5 tertiary centers in 3 countries in South East Asia was conducted. Details of training and clinical outcomes of ESD cases, with follow-up data from these centers, were analyzed.* Results.* Seven endoscopists from the 5 centers performed a total of 35 cases of ESD in the upper gastrointestinal tract (UGIT) over a 6-year duration. Details of the lesions excised were as follows: median size was 20 mm, morphologically 20 (68.6%) were flat/depressed and 6 (17.1%) were submucosal, and histologically 27 (77.1%) were neoplastic. The median duration of ESD procedures was 105 minutes, with an en-bloc resection rate of 91.4%. There was 1 (2.9%) case of delayed bleeding, but no perforation nor mortality in any of the cases. The recurrence rate after ESD was 5.7%. A prolonged ESD duration was influenced by a larger size of lesion (25 mm, *p* = 0.02) but not by factors related to the training experience of endoscopists.* Conclusions.* ESD in the UGIT is feasible and safe in low volume centers in Asia.

## 1. Introduction

Endoscopic resection is the least invasive and cost-effective way of treating superficial malignant lesions in the digestive tract. Endoscopic submucosal dissection (ESD) has now become the accepted technique of resection of early tumors in the upper gastrointestinal (GI) tract, although it has long been practiced among Japanese endoscopists who pioneered this technique. ESD, however, is technically challenging and is rarely practiced outside of Japan or Korea [1]. A steep learning curve to master ESD, prolonged procedure duration, increased procedure risks, lack of commensurate reimbursement, and need for specialized tools have become challenges in mastering ESD [2]. Endoscopic mucosal resection (EMR) is technically less demanding and seen by many as an alternate method of endoscopic resection for early GI neoplasia. However, EMR is unable to achieve en-bloc resection for lesions >20 mm in size and has been shown to have a higher rate of tumor recurrence compared to ESD.

In Japan, several experts have suggested that competency in ESD can only be achieved following supervised performance of between 30 and 80 ESD cases [3]. Such a volume of ESD cases may be easily achieved in a short space of time in Japan and Korea, where a high incidence of early gastric cancer detection resulting from an active screening programme is present [4, 5], but this is clearly not the case elsewhere in Asia or in the West [6]. This does not necessarily indicate then that ESD should not be practiced outside of East Asia, as there is a growing indication for ESD beyond early gastric malignancy, such as for submucosal GI tumors [7–9]. As such, recent experts from the West have proposed a variation to the Japanese recommendation for training in ESD. In addition to being experienced in general therapeutic GI endoscopy, Draganov et al. have suggested that proficiency in conventional EMR, together with animal model training, may be sufficient for endoscopists in the West to embark on ESD in humans [10].

The incidence of gastric cancer in South East Asia is low at 10.1 per 100,000 populations (age-standardised rates) [11]. Nevertheless, with its moderate/low prevalence of* H. pylori* infection, early gastric cancer is not totally unrecognized in the Southeast Asian region [12]. Recognizing the need to develop ESD within their respective countries, several endoscopic centers in South East Asia, namely, in Thailand, Singapore, and Malaysia, had embarked on ESD for upper GI tumors over the last 6 years. Specific individuals in each center, usually with a background of EMR experience, underwent training on animal models and performed ESD under supervision by experts before embarking on the procedure independently. This report aims to document the collective feasibility and safety of ESD in the main centers performing ESD in South East Asia.

## 2. Methods

ESD in the upper GI tract had been commenced in the following 5 centers in South East Asia over the past several years: Chulalongkorn University Hospital and Rajavithi Hospital in Bangkok; National University Hospital and Singapore General Hospital in Singapore; and University Malaya Medical Center in Kuala Lumpur. Prospective data on experience of ESD operators, procedure details, and clinical follow-up information were obtained in all cases using a structured clinical record form.

Data on ESD in the upper GI tract alone were collected in this study, as mucosal or submucosal lesions in the esophagus and stomach were the commonest indication for ESD in all centers.

### 2.1. ESD Technique

Prior to ESD, all gastric lesions were carefully evaluated with chromoendoscopy and endoscopic ultrasound (EUS) where indicated. The endoscopic appearances of mucosal lesions were classified based on the Paris Classification of superficial tumors [13]. Tissue biopsies were taken in cases prior to ESD where indicated. ESD was performed in a standard manner [14]. All patients had sedation, which was administered by an anaesthetist. After chromoendoscopy with 0.2% Indigo carmine dye to delineate the border of the tumor, circumferential marking with a needle knife (KD-1L-1, Olympus, Tokyo, Japan) or ERBE hybrid knife (Erbe Elektromedizin GmbH, Tuebingen, Germany) was made. Submucosal injection was performed using a standard solution (Gelafundin or normal saline with Adrenaline and Indigo carmine in a ratio of 20 : 1 : 1) [15]. After an adequate submucosal lift of the periphery of the tumor was made, an initial mucosal incision was performed (needle knife KD-1L-1, Olympus, Tokyo, Japan, or ERBE hybrid knife). This was followed by a circumferential incision and subsequent submucosal dissection using the IT2 knife, Dual knife (Olympus, Tokyo, Japan) or ERBE hybrid knife (Erbe Elektromedizin GmbH, Tuebingen, Germany), with repeated submucosal injection where necessary. Following en-bloc resection of the tumor, the mucosal defect was carefully inspected and areas of bleeding or prominent vessels were cauterized using hemostatic forceps (Olympus, Tokyo, Japan). After ESD, all patients had a routine follow-up endoscopic examination at 1 month to check for ulcer healing and subsequently at 6 and 12 months. Further assessment was made depending on the findings at repeat endoscopy.

### 2.2. Clinical Data Parameters

All resected specimens were examined histologically based on the Vienna Classification [16]. Involvement or clearance of the margins was recorded. All patients' vital signs were monitored after procedure. Hemoglobin levels were checked on the day after the procedure routinely and were clinically indicated. Procedure duration was described from the time the endoscope entered the oral cavity until the time it was removed from the patients' mouth. Complications were divided into intraprocedure and postprocedure. These included immediate and delayed bleeding, perforation, and complications associated with sedation.

Patients who had a reduction in hemoglobin by 2 g/dL or requiring blood transfusion after procedure were labeled as delayed bleeding. All patients were followed up for a median period of 24 weeks. The endoscopic findings during follow-up were recorded. “Local recurrence” was defined as the presence of tumor after 2 negative gastroscopy follow-up examinations.

### 2.3. Main Outcomes

The main outcomes of this study were to examine the feasibility and safety of ESD in low volume centers. Feasibility will be assessed with the following parameters: duration of ESD procedure, completeness of tissue resection (by histological assessment), and recurrence rate at follow-up endoscopy. The safety of ESD will be based on the rates of immediate and delayed complications after ESD. Comparisons with published data on ESD in both European and Eastern (Korean and Japanese) series will be explored.

### 2.4. Statistical Analysis

All raw data obtained from each center were recorded and analyzed using a standard software package (SPSS version 21, Chicago, IL). Mean and median values of variables were calculated and presented accordingly. Basic analyses on predictors of ESD technical success and complications will be performed. Statistical significance was assumed at a *p* value of <0.05.

## 3. Results

### 3.1. Endoscopists

A total of 7 endoscopists from the 5 centers were involved in this study: Thailand *n* = 3, Singapore *n* = 2, and Malaysia *n* = 2. The median general endoscopy experience was 17 (range 5–22) years and all endoscopists had prior EMR experience before embarking on ESD (median 11 years, range 3–15 years). All 7 endoscopists had undergone animal model training for ESD and 3 of them were trained in Japan. A median of 25 ESD cases (min 15, max 40) had been observed prior to commencing ESD individually in patients.

### 3.2. Case Description

**Figure 1:**
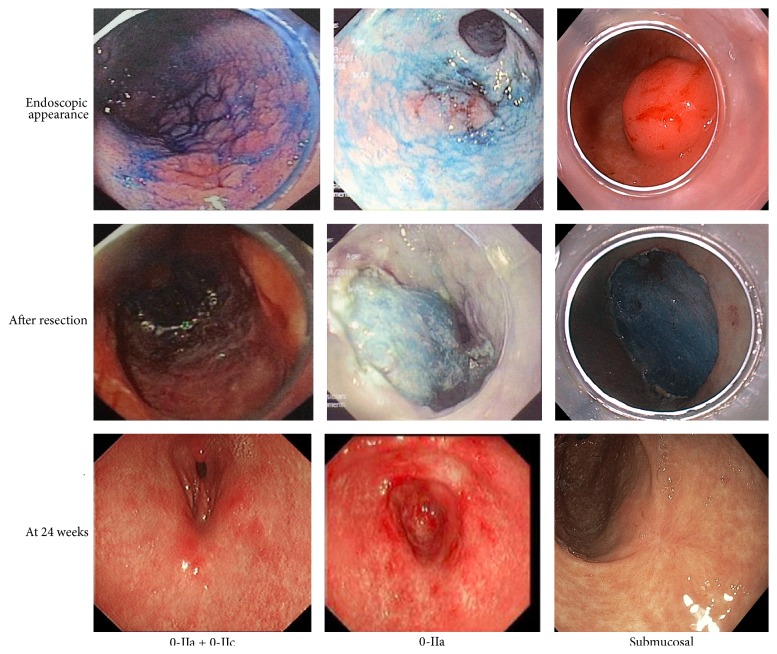
Endoscopic images of various lesions excised by ESD in this series.

A total of 35 patients had undergone ESD between 2009 and 2015. 18 (51.4%) cases were female and the median age was 71 (range 21–85) years. Details of the lesions are summarized in Table 1. The majority of lesions identified were in the lower stomach (54.3%). There were a range of lesions morphologically, with Type 0-IIa being the commonest; examples of some of these lesions are shown in Figure 1. ESD was performed for 6 submucosal lesions, as they were suspected neuroendocrine tumors (NET). The median size of lesions was 20 mm (IQR 15–25 mm), with a range from 5 mm to 60 mm. Based on histological evaluation of the resected specimens (Table 1), the lesions were subsequently categorized as neoplastic (mucosal dysplasia, neoplasia, or NET) *n* = 27 (77.1%) and benign mucosal/benign submucosal lesions *n* = 8 (22.9%).

### 3.3. ESD Feasibility

**Figure 2:**
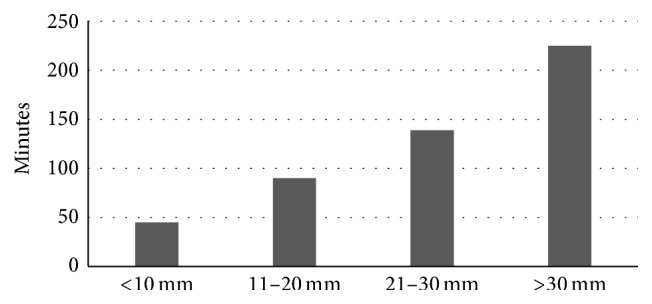
Duration of ESD procedure in relation to the size of lesion.

23 (65.7%) cases were performed under general anaesthesia, 9 (25.7%) cases with Propofol sedation, and 3 (8.6%) cases with Midazolam sedation alone. The median duration of ESD procedures was 105 IQR (65–184) minutes, with a range from 15 to 480 minutes.  Figure 2 illustrates the linear relationship between lesion size and the median duration of ESD procedures.

En-bloc resection was successful in 32/35 lesions (91.4%) and the remaining 3 lesions were resected in a piecemeal manner. From en-bloc resected specimens, histologically, 30/35 (85.7%) specimens showed complete resection (R0) and 2/35 (5.7%) samples had R1 resection (see Table 2). Six patients had histology suggesting incomplete margin clearance (duodenal NET *n* = 1, gastric NET *n* = 1, and gastric adenocarcinoma *n* = 4) and were referred for surgical resection.

### 3.4. ESD Safety

Significant profuse bleeding was reported in only 1 (2.9%) case, but this was successfully treated with hemoclips. All minor bleeding during ESD was managed immediately with either hemostatic forceps or hemoclips. No patient required a blood transfusion during or 24 hours after procedure. There were no perforations and no immediate mortality resulting from ESD. No complications relating to sedation/anaesthesia, such as hypoxia or aspiration, were observed in this series.

### 3.5. Follow-Up

All patients were followed-up for a median duration of 24 weeks (range 4–224 weeks). Recurrence was detected in 2/35 (5.7%) cases—1 with adenocarcinoma (en bloc, R1) and the other with a gastric NET (en bloc, R0). EMR was performed for the adenocarcinoma recurrence with complete marginal clearance. The gastric NET recurrence is being monitored without immediate plans for resection as the original histology was a low-grade NET.

### 3.6. Predictors of ESD Outcomes

Due to the low number of complications and high number of en-bloc resections, predictors of these outcomes could not be analyzed in this study. “Procedure duration” was therefore evaluated as a proxy of ESD outcomes in this study. The median duration of ESD was 105 minutes and procedure duration beyond this was defined as a poorer outcome. Factors which may have influenced procedure duration were explored by univariate analysis (Table 3). Factors related to training (duration of EMR experience, number of ESD cases observed, and prior experience in Japan) did not influence ESD procedure duration. The main predictor for a prolonged ESD procedure was the size of the lesion (25 mm versus 15 mm, *p* = 0.02).

## 4. Discussion

Most publications of ESD case series have been derived from high volume centers in Japan and Korea [17, 18], but reports from Western countries and smaller Asian nations are gradually emerging. A recent nationwide survey from 14 centers in France performing ESD in the UGIT reported a mean procedure duration of 108.2 ± 62 (range of 37 to 330) minutes, en-bloc resection, and R0 rate 91.7% and 71.2%, respectively [19]. In Taiwan, a multicenter case series reported a median procedure duration of gastric ESD of 92.4 minutes for lesions of a median size of 18 mm, with an en-bloc resection rate of 91% [8]. In this multicenter, multinational case series from South East Asia, the median procedure duration of 105 minutes for lesions with a median size of 20 mm, with an RO resection rate of 85.7%, compares favourably to these published reports from non-Japanese/Korean centers.  Table 4 is a brief summary comparing ESD experiences from low volume centers from different regions.

There were no major complications from this case series, probably due to the smaller number of cases. Perforations complicating ESD have been reported to range from 1.2% to 5.2% and delayed bleeding from 0% to 15.6% [20]. However, the <100% en-bloc resection rate, with tumor recurrence, was probable indicator of operator inexperience. In our series, 19 (54.3%) lesions located in the lower portion of the stomach had a successful en-bloc resection, except for a single lesion. Location of a lesion in the upper part of stomach, previous scars, and an undifferentiated pathologic type have been identified as risk factors for not achieving an en-bloc resection [18]. Local recurrence occurred in 2 (5.7%) cases in this series. A previous meta-analysis by Lian et al. has suggested that tumor recurrence is much lower for ESD when compared to EMR [17]. However rates of tumor recurrence post ESD have been shown to vary from 2% to 35% [21]. Piecemeal resection and resection margin involvement by tumor are two main reasons for recurrence after ESD. En-bloc resection is the single most important factor for a curative ESD without recurrence, which can give a disease-specific 5-year overall survival of 97.1%–100% [22]. Six patients who had incomplete histological clearance had subsequent surgery. However, no residual tumor cells were present in surgically resected specimens. It is likely that the ESD had actually been successful, but histology had been inaccurate or cautery effect at the resection margin may have ablated tumor outside the resected specimen.

A prolonged duration of ESD can lead to unwanted complications resulting from sedative or anaesthetic medications. Furthermore, many of the patients requiring an ESD procedure are elderly (median age 71 years in this case series), with the potential of cardiorespiratory complications with sedation/anaesthesia. Hence, a prolonged duration of procedure has been accepted as an undesirable outcome of ESD [18]. In a large retrospective series of 1000 ESD cases from Korea, Chung et al. defined >60 minutes as a prolonged ESD duration and identified large size of lesion, upper stomach location of tumor, presence of scar, recurrent lesion, and flat macroscopic morphology as predictors [18]. Of these factors, large size of lesion (OR 4.5) and recurrent lesion (OR 3.0) were the most predictive of a prolonged duration of procedure. In our small series, we similarly identified that a larger size of lesion, but no other characteristics, influenced the duration of ESD procedure. It is likely that the small sample size in this series prohibited an accurate assessment of the other factors other than size, that is, Type 2 statistical error.

ESD was initially developed for en-bloc resection of mucosal tumors. Due to its technical success and an improvement in accessories which can be used for closure of transmural perforations, ESD has expanded to include resection of various submucosal tumors in the stomach. An initial study by Li et al. on 29 neuroendocrine tumors showed that 28 lesions (96.6%) in the upper GI tract could be completely resected, with only 1 case of delayed bleeding, 0 perforations, and 1 recurrence [9]. Several other case series have recently reported successful ESD for various gastric submucosal tumors such as ectopic pancreas, ectopic spleen, leiomyoma, and gastrointestinal stromal tumor (GIST) [23, 24]. In another case series from France, the authors reported successful resection of 33 submucosal lesions in the stomach for lesions <20 mm, with the majority of lesions being resected by ESD [25]. In this study, 6 lesions dissected were submucosal in origin. All lesions were resected safely without any complications, indicating that ESD for these deeper lesions was feasible even in low volume centers.

Data from this case series may have been limited by the self-reporting method of individual endoscopists. Nevertheless, the modest records of procedure duration are indicative of ESD being performed in a low volume setting. The recent large series of ESD in multiple centers in France [19] and a prior study in Nagano [26] suggest that center volume may not necessarily be the sole factor affecting outcomes in ESD. Unlike young Japanese endoscopists who embark on ESD, endoscopists here develop a significant amount of experience in therapeutic endoscopy prior to performing ESD. In particular, standard EMR for lesions in the GI tract share many technical aspects with ESD, namely, chromoendoscopy, submucosal elevation prior to resection, and use of electrocautery devices and that of clips for closure of mucosal defects after resection. We concur with a recent review article which suggested that ESD can be taken up by non-Japanese endoscopists who were proficient in conventional EMR and had undergone sufficient training in animal models [10]. Although EGC may not be common in this region, the expanding role of ESD as a minimally invasive therapeutic modality for gastric submucosal tumors and other such lesions indicates that there is still a need for developing this skill among endoscopists in this region.

## Figures and Tables

**Table 1:** Clinical characteristics of ESD lesions (*n* = 35).

Location	
Esophagus	3 (8.6%)
Upper stomach	4 (11.4%)
Mid stomach	8 (22.9%)
Lower stomach	19 (54.3%)
Duodenum	1 (2.9%)
Morphology	
0-I	4 (11.4%)
0-Is	1 (2.9%)
0-IIa	12 (34.3%)
0-IIc	4 (11.4%)
0-IIa + 0-IIc	8 (22.9%)
Submucosal	6 (17.1%)
Size	Median 20 (range 5–60) mm
Histology	
Adenocarcinoma	17 (45.8%)
Severe dysplasia	6 (17.1%)
Intestinal metaplasia	1 (2.9%)
Neuroendocrine tumor	3 (8.6%)
Leiomyoma	3 (8.6%)
Ectopic pancreatic tissue	2 (5.7%)
Hyperplastic	1 (2.9%)
Lipoma	2 (5.7%)

**Table 2:** Outcomes of ESD (*n* = 35 cases).

Median duration (minutes)	105 (15–480)
Complete resection	
En bloc resection	32 (91.4%)
R0 resection (includes en bloc and piecemeal specimens)	29 (82.9%)
En bloc with R0 resection	27 (77.1%)
Complications	
Delayed bleeding	1 (2.9%)
Perforations	0
Recurrence	
Recurrence	2 (9.8%)

**Table 3:** Predictive factors for a prolonged ESD duration.

	Prolonged ESD duration (>105 mins)		*p*
	Yes (*n* = 17)	No (*n* = 18)	
Cases done by endoscopists with training in Japan (total *n* = 16)	9 (56.3%)	7 (43.7%)	0.4^#^
Endoscopy training (median years)	18	17	0.62^*∗*^
ESD cases observed (median number)	30	40	0.06^*∗*^
Previous EMR experience (median years)	11	9.5	0.40^*∗*^
Location of lesion			
Lower stomach	4 (21.1%)	15 (78.9%)	0.29^#^
Non-lower stomach	6 (37.5%)	10 (62.5%)	
Lesion morphology			
Flat/depressed (0-IIc)	8 (42.1%)	11 (58.9%)	0.51^#^
Elevated (0-IIa)	9 (56.3%)	7 (44.7%)	
Size of lesion (median mm)	25	15	0.02^*∗*^

^*∗*^Mann–Whitney *U* test.

^#^Chi-square test.

**Table 4:** Summary of ESD in upper GI tract outcomes from low-volume centres in the world.

	Taiwan [8]	Italy [23]	Portugal [27]	France [19]	South East Asia (this study)
Year	2004–2007	2005–2011	2005–2008	2010–2013	2009–2015
Centers	8	2	1	14	5
Study method	Retrospective	Retrospective	Retrospective	Prospective	Retrospective
Number of ESD cases	70	20	19	177 (upper GI only)	35
Size (mm)	Mean 32.6 ± 13.9	Median 29 (15–60)	Median 25 (15–30)	Mean 39 ± 23	Median 20 (5–60)
Procedure time (minutes)	Median 92.4 (25–210)	Medial 119.1 (40–240)	Median 90 (40–300)	Mean 108.2 ± 62	Median 105 (15–480)
Knife used	IT knife Needle knife	IT knife Hook knife	IT knife Hook knife	Dual knife Flush knife	IT knife Hybrid knife
Outcomes					
En bloc resection, *n* (%)	64/70 (91.4%)	—	15/19 (79%)	292/319 (91.5%)	32/35 (91.4%)
R0 en bloc resection, *n* (%)	NA	18/20 (90%)	13/19 (68%)	277/319 (71.2%)	27/32 (77.1%)
Complications					
Bleeding, *n* (%)	4/70 (5.7%)	0	1/19 (5.3%)	15 (4.7%)	1 (2.9%)
Perforation, *n* (%)	3/70 (4.3%)	3/20 (15%)	0	26 (8.1%)	0
Recurrence					
Esophagus, *n* (%)	—	NA	—	4/60 (6.7%)	0
Stomach, *n* (%)	2/70 2.8%	NA	0	5/63 (6.1%)	2/35 (9.8%)

ESD: Endoscopic submucosal dissection; R0: complete resection with no margin involvement; NA: not available; and IT: insulated tip.
